# Mikulicz’s disease combined with IgG4-related hypophysitis: a case report

**DOI:** 10.1186/s12877-024-05142-7

**Published:** 2024-06-16

**Authors:** Shu-Fan Zhang, Jing Deng, Jie Xiao, Bi-Hua Wu

**Affiliations:** Department of Geriatrics, The Affiliated Hospital of North Sichuan Medical College, Nanchong, Sichuan 637000 China

**Keywords:** IgG4-related disease, Mikulicz’s disease, IgG4-related hypophysitis, Pituitary stalk, Case report

## Abstract

**Background:**

IgG4-related diseases are very uncommon, and its diagnosis and treatment are complicated as it encompasses multiple disciplines.

**Case presentation:**

: A 77-year-old woman was admitted with a jaw mass and nausea and vomiting. Laboratory tests showed elevated serum IgG4, pituitary MRI suggested thickening of the pituitary stalk, and head and neck CT suggested orbital and mandibular masses. Patients with mandibular mass were diagnosed with Mikulicz’s disease with IgG4-related hypophysitis. We found no other evidence of causing thickening of the pituitary stalk. She was given oral prednisolone 30 mg daily, and her nausea and vomiting improved significantly, and the mandibular and ocular masses decreased in size.

**Conclusion:**

Mikulicz’s disease combined with IgG4-related hypophysitis is a rare case of IgG4-RD in elderly women. IgG4-RD is one of the causes of head and neck exocrine gland mass and pituitary stalk thickening in the elderly.

## Background

IgG4-related disease (IgG4-RD) is a chronic fibroinflammatory disease characterized by enlargement of the involved organs, elevated serum IgG4 levels, and a large infiltration of IgG4-positive plasma cells in the involved organs [1].When the lacrimal and salivary glands are involved, it is known as Mikulicz’s disease(MD).MD is prevalent in females with high serum IgG4 levels [2–4].Pituitary involvement may present as IgG4-related hypophysitis (IgG4-RH). Gender disparities exist in IgG4-RH manifestation: men commonly affected in later years with IgG4-related disease (IgG4-RD) and elevated IgG4; women typically present earlier with solitary pituitary lesion, lower IgG4, and frequent autoimmunity [5, 6].Previously reported MD complicated with IgG4-RH were male patients, and most of them were complicated with other types of IgG4-RD or autoimmune diseases [7–9]. We report a patient diagnosed MD complicated with IgG4-RH, an elderly woman, which has not been reported before. Our patients have no previous history of autoimmune disease, and the main manifestation of pituitary involvement is the impairment of gonadal axis function, which is different from the previous reports of total adenohypophysis dysfunction, and is easy to be omitted for patients with concealed clinical manifestations.

## Case presentation

**Fig. 1:**
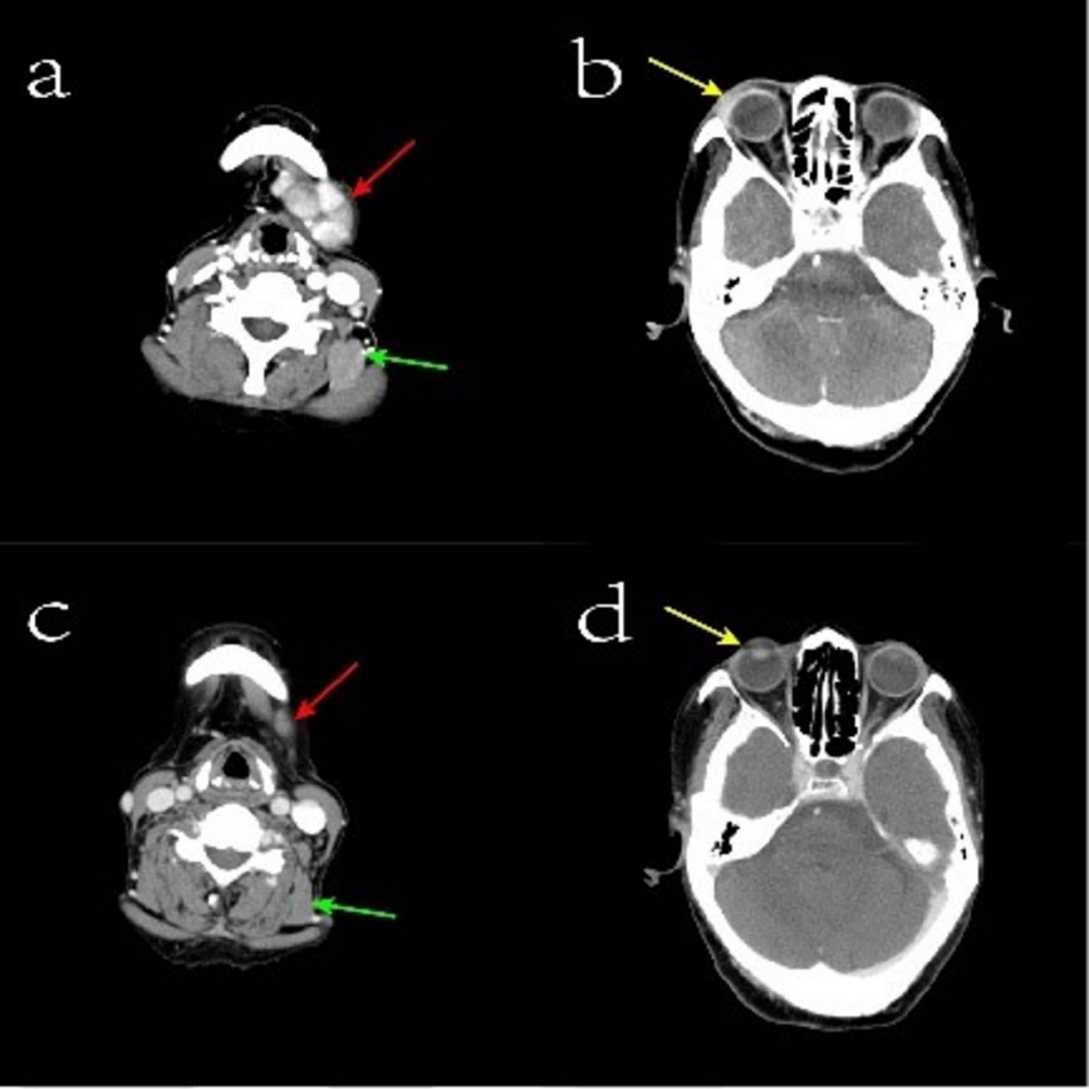
Enhanced computerized tomography image of the head and neck. Figure **a**: The red arrow indicates the mandibular mass before hormonal treatment, and the green arrow indicates the involved supraspinatus muscle before treatment; Figure **b**: The yellow arrow refers to the orbital tissues enhanced and strengthened before treatment; Figure **c**: The red arrows show the reduced submandibular mass after hormonal treatment, and the green arrows refer to the normalized supraspinatus muscle after treatment. Figure **d**: The yellow arrows refer to the orbital tissues after treatment, with no significant enhancement shadow

**Fig. 2:**
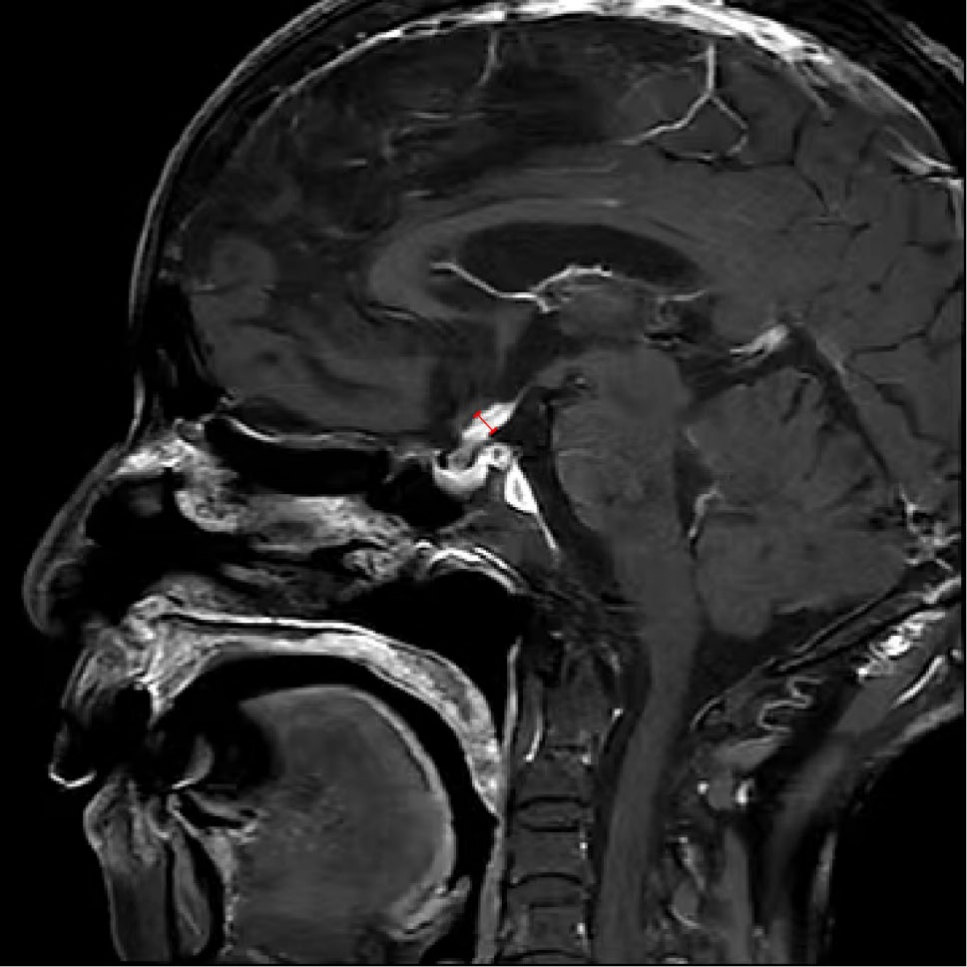
Magnetic resonance image of the pituitary gland. The T1 sagittal fat suppression sequence shows a thickened pituitary stalk with the widest point being 5.6 mm

**Fig. 3:**
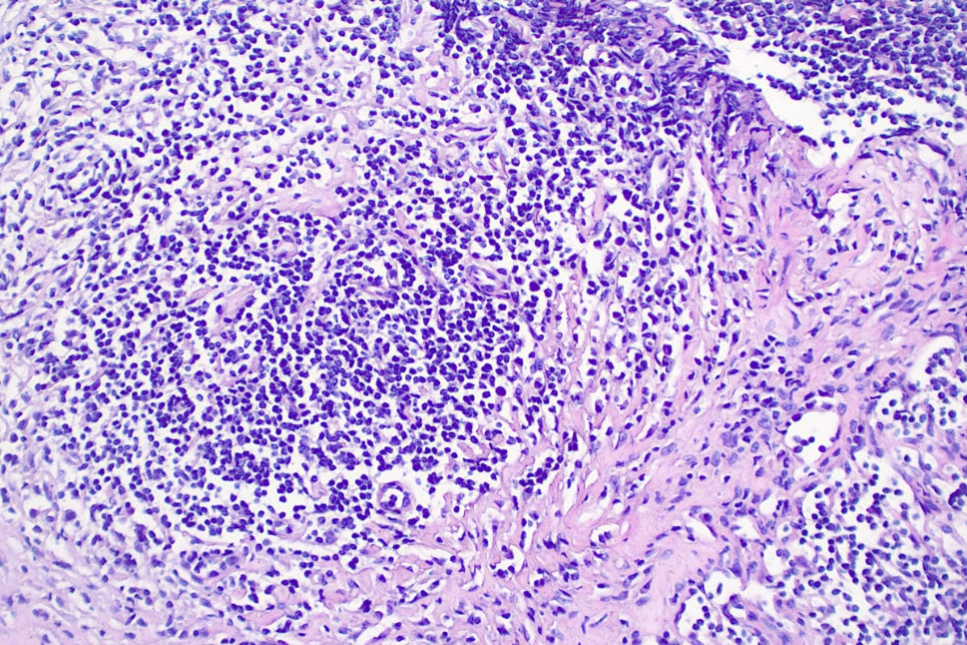
Biopsied tissue of the submandibular mass (HE staining, x200) proliferative fibrous tissue and lymphocytic infiltration are observed in the tissue

**Fig. 4:**
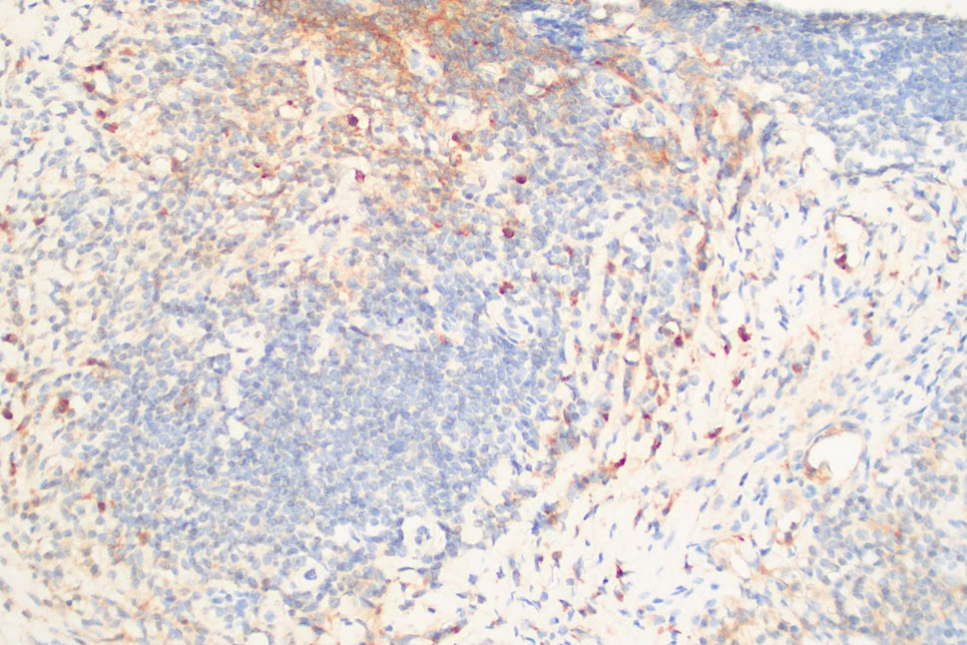
A sample viewed at 200x magnification using IgG4 immunohistochemistry. A large number of plasma cells and IgG4-positive plasma cells > 50 per high-power field

A 77-year-old woman was sent to the hospital because of the discovery of a submandibular mass that had been progressively growing for more than six years, and recurrent dry heaving for two months nausea, vomiting, depression and painful were also obvious. Prior to presentation, she was admitted to the Department of Oncology and the maxillofacial surgery department. They suggested an exploratory resection of the left submandibular gland mass. However, the surgery was not performed because the patient experienced repeated vomiting. Medical examination on admission: The mass was under the left mandible, movable and mildly painful on pressure. Further investigations showed no additional abnormalities. Laboratory examinations including complete blood count, liver and kidney function tests, coagulation function and levels of carcinoembryonic antigen and other routine tests showed no obvious abnormalities. Hormonal evaluation revealed a follicle-stimulating hormone(FSH) level of 0.31 mIU/mL (normal range 23.00–116.30 mIU/mL) and a luteinizing hormone(LH) level of 0.00 mIU/mL (normal range 15.90–54.00 mIU/mL). The levels of adrenocorticotropic hormone (ACTH) and thyroid-stimulating hormone (TSH) were within normal limits. Hormone stimulation test against only gonadotrophins gave no reaction. In terms of connective tissue-related antibodies, the patient tested positive for antinuclear antibodies at a titer of 1:100. Immunoglobulin assessment indicated a significantly elevated IgG4 level of 6.680 g/L (normal range 0.030–2.010 g/L), with a subsequent reading of 7.980 g/L. Computed tomography (CT) of the neck and head revealed enlarged left submandibular gland, submandibular region, sub-chin region, left scapular raphe muscle and the right lacrimal region (Fig. 1a and b). Magnetic Resonance Imaging (MRI) of the pituitary gland showed enlargement of pituitary gland and pituitary stalk (Fig. 2). CT of the abdomen did not show any abnormality in the pancreas, liver, or retroperitoneal tissue. A puncture biopsy of the submandibular gland mass suggested proliferative fibrous tissue and lymphocytic infiltration in the left submandibular nodule (Fig. 3). Immunohistochemistry analysis showed multiple cells positive for IgG4 (Fig. 4). According to The 2019 American College of Rheumatology–European Union against Rheumatism Classification criteria for IgG4-RD of rheumatism, this patient obtained the total score of 31 points [1], a diagnosis of IgG4-related MD with associated pituitary inflammation was established. The patient was started on a treatment regimen with prednisone acetate at a dosage of 30 mg/d, also take calcium carbonate D3 to prevent osteoporosis. After one month, the dose was reduced to 15 mg/d. After two months of treatment, a follow-up CT of the neck suggested significant reduction of mass in the left submandibular gland, submandibular region, sub-chin region, the supraspinatus muscle and the right lacrimal region (Fig. 1c and d). The patient’s symptoms of vomiting and mental status improved significantly compared to the initial presentation. She was subsequently regularly followed up on an outpatient basis.

## Discussion and conclusion

IgG4-RD is a rare, systemic, immune-mediated disease first identified in elderly Japanese men at the beginning of the 21st century [10], and the disease was officially named in 2010 [11]. Antigen presentation by B cells, induction of differentiation by Th2 cell-dominant B cells, and the pathogenic role of CD 4 + CTL in ultimately causing tissue fibrosis are the possible mechanisms of IgG4-RD [12–15]。.

The clinical manifestations of IgG4-RD show gender disparities, and Wang et al. [16] discovered significant gender differences in the affected organs in patients aged ≥ 55 years old, with superficial organ involvement (e.g., MD and thyroiditis) being more common in female patients and visceral organ involvement (autoimmune pancreatitis, sclerosing cholangitis, and retroperitoneal fibrosis) being more common in male patients. IgG4-RD rarely affects a single organ, and about 58-88% of patients have multiple organ involvement. When the pituitary gland is involved, it usually presents as pituitary inflammation. The clinical manifestations of IgG4-RH vary depending on the site of involvement. Symptoms associated with hypopituitarism (e.g., vomiting, nausea, weight loss, fever, loss of appetite, and loss of libido) may be seen when the anterior lobe of the pituitary gland is involved. Involvement of the posterior lobe of the pituitary gland can lead to urolithiasis [5]. Moreover, a thickened pituitary stalk and pituitary gland can compress the optic nerve or optic chiasma, resulting in decreased visual acuity, altered color vision, and visual field defects, which can occur independently or simultaneously with other organ involvements [17].The diagnosis of IgG4-RD should be based on a combination of typical histopathological features, clinical signs and symptoms, serological findings, and imaging manifestations.

In 2019, with the support of the American College of Rheumatology and the European Union of Rheumatology, a global panel of experts in IgG4-RD developed a comprehensive classification system [1]. Involvement of multiple glands account for a large proportion of this classification system. Both SS and MD may present with marked enlargement of the parotid, submandibular, and lacrimal glands. In 1953, Morgan et al. [18] reported that MD and SS were pathologically identical and considered a subtype of SS. However, further research by Yamamoto et al. [19] revealed that MD showed high serum IgG4 levels and IgG4 + plasma cell infiltration in the lacrimal and salivary gland tissues, whereas SS did not have these pathological findings, suggesting that the two are different diseases. SS and MD are differentiated based on their typical histopathology, laboratory tests, and clinical features. MD is usually characterized by bilateral, symmetrical, and painless swelling, and unlike SS, symptoms such as dry mouth, dry eyes, or arthralgia are rare. In our case, the symptoms of dry mouth and dry eyes were not obvious, which was different from that of SS, the serological IgG4 was elevated, and the histopathology of mandible suggested that the plasma cell infiltration of IgG4 + supported the diagnosis of MD. The change of pituitary gland is an important criterion in the diagnostic criteria of IgG4-RH. The elderly patient mainly presents with pituitary morphological abnormalities characterized by thickened of the pituitary stalk, which raises suspicion of a malignant tumor. However, previous studies have indicated that pituitary stalk thickness with loss of posterior MRI signal cannot differentiate whether metastatic lesions are caused by systemic diseases [20]. Linking clinical characteristics with the absence of systemic clinical and biological signs help discern whether a pituitary lesion needs more thorough workup to rule out metastasis from a distant primary [21]. The patient presents no clinical symptoms of malignant tumors and both whole-body CT and serological examinations show no evidence of tumors. Gutenber et al. [22]developed a clinic-radiological scoring system that can effectively differentiate between pituitary inflammation and pituitary adenoma. The patient scored − 4 in the system, indicating a diagnosis of pituitary inflammation.

Currently, glucocorticoids (GCs) remain the first-line treatment for IgG4-RD. In our cases, prednisone 30 mg/d was administered. After 2 months, We observed the reduction of the mass and the disappearance of symptoms such as nausea and vomiting, indicating that glucocorticoid therapy may effective for this patient. However, for patients with high disease activity or a high risk of relapse, a tapering of GCs is recommended, followed by maintenance therapy with disease-modifying antirheumatic drugs (DMARDs). Predictors of relapse after treatment can include baseline plasma cell counts in serum, serum IgG4 levels at initial treatment, serum IgG4 levels in response to treatment, and the results of 18 F-FDG PET/CT scans. Initial serum IgG4 levels are primarily used to predict the risk of relapse after treatment with DMARDs, whereas changes in serum IgG4 levels in response to treatment can be evaluated in terms of the risk of relapse when treated with GCs alone or in combination with DMARDs [23–26]. The diagnosis of the patient in the current case was confirmed by the improvement of clinical symptoms, serologic IgG4 levels, and imaging manifestations after treatment with GCs, compared to pretreatment status. For patients who experience relapse, a combination therapy with DMARDs is recommended. Some studies have found that the combination of GCs with low-dose rituximab is more effective and safer in the treatment of IgG4-RD compared to a combination of GCs and leflunomide [27]. Rituximab has also been found to be important in the remission of IgG4-RD and in maintaining its stability. However, the treatment has some economic limitations [28];therefore, a multifaceted assessment is needed when choosing a treatment regimen.

In summary, we report an elderly female patient with Mikulicz’s disease complicated with IgG4-associated hypophysitis. IgG4-RD is one of the causes of head and neck exocrine gland mass and pituitary stalk thickening in the elderly.

## Data Availability

The data used to support the findings of this study are included within the article.

## Abbreviations

IgG4-RD: IgG4-related diseases
IgG4-RH: IgG4-related hypophysitis
MD: Mikulicz’s disease
FSH: Follicle-stimulating hormone
LH: Luteinizing hormone
ACTH: Adrenocorticotropic hormone
TSH: Thyroid-stimulating hormone
CT: Computed tomography
MRI: Magnetic Resonance Imaging
SS: Sjogren’s syndrome
GCs: Glucocorticoids
DMARDs: Disease-modifying antirheumatic drugs. FDG: fluorodeoxyglucose
PET/CT: Positron emission tomography/computed tomography
